# GWAS for the identification of introgressed candidate genes of *Sinapis alba* with increased branching numbers in backcross lines of the allohexaploid *Brassica*

**DOI:** 10.3389/fpls.2024.1381387

**Published:** 2024-06-24

**Authors:** Kaushal Pratap Singh, Preetesh Kumari, Pramod Kumar Rai

**Affiliations:** ^1^ Plant Protection Unit, Indian Council of Agricultural Research (ICAR)-Directorate of Rapeseed Mustard Research, Sewar, Bharatpur, India; ^2^ Genetics Division, ICAR-Indian Agricultural Research Institute, New Delhi, India; ^3^ School of Agriculture, Sanskriti University, Mathura - Delhi Highway, Chhata, Mathura, India

**Keywords:** *Brassica juncea*, backcross introgression lines (BCILs), plant architecture, genome-specific SSRs, association mapping, trait-linked SSRs, candidate genes

## Abstract

Plant architecture is a crucial determinant of crop yield. The number of primary (PB) and secondary branches (SB) is particularly significant in shaping the architecture of Indian mustard. In this study, we analyzed a panel of 86 backcross introgression lines (BCILs) derived from the first stable allohexaploid Brassicas with 170 *Sinapis alba* genome-specific SSR markers to identify associated markers with higher PB and SB through association mapping. The structure analysis revealed three subpopulations, i.e., P1, P2, and P3, in the association panel containing a total of 11, 33, and 42 BCILs, respectively. We identified five novel SSR markers linked to higher PB and SB. Subsequently, we explored the 20 kb up- and downstream regions of these SSR markers to predict candidate genes for improved branching and annotated them through BLASTN. As a result, we predicted 47 complete genes within the 40 kb regions of all trait-linked markers, among which 35 were identified as candidate genes for higher PB and SB numbers in BCILs. These candidate genes were orthologous to *ANT*, *RAMOSUS*, *RAX*, *MAX*, *MP*, *SEU*, *REV*, etc., branching genes. The remaining 12 genes were annotated for additional roles using BLASTP with protein databases. This study identified five novel *S. alba* genome-specific SSR markers associated with increased PB and SB, as well as 35 candidate genes contributing to plant architecture through improved branching numbers. To the best of our knowledge, this is the first report of introgressive genes for higher branching numbers in *B. juncea* from *S. alba*.

## Introduction

Crop wild relatives (CWRs) have been established as valuable sources for incorporating genes for adaptation in crop plants under changing climatic conditions by allowing modifications in crop genetic constitution and developing high-yielding crop varieties (Hajjar and Hodgkin, 2007; Bohra et al., 2022). Among CWRs, Brassica wild relatives (BWRs) have maintained valuable genetic diversity for improving yield-related and climate-resilient traits, such as plant vigor, architecture, and the ability to withstand biotic and abiotic stresses. By enriching the gene pool of crop species, BWRs have contributed significantly by augmenting the genetic diversity of cultivated plants (Quezada-Martinez et al., 2021). The family Brassicaceae includes several wild genera, such as *Brassica fruticulosa*, *Camelina sativa*, *Diplotaxis catholica*, *D. erucoides*, *Erucastrum abyssinicum*, *Erucastrum cardaminoides*, *Eruca sativa*, *Moricandia arvensis*, and *Sinapis alba*. Among these, *S. alba* (white mustard), commonly grown as a condiment, has the highest number of PB and SB (Kumari et al., 2011; Singh et al., 2023). Interestingly, *S. alba* is diploid (2n= 24, SS) and has close genetic proximity to *Brassica nigra* L. (BB, 2n=16), a diploid ancestor of the Indian mustard (Warwick and Black, 1991).

Indian mustard (*B. juncea*; AABB; 2n=36) is an important oilseed crop cultivated globally for its edible oil, vegetables, and condiments (Banga and Banga, 2016). The genetic diversity of Brassica oilseed crops has become limited due to strong selection pressure for yield and quality attributes, which has made it increasingly challenging to further increase yields. Consequently, the production levels of these crops have plateaued (Singh et al., 2021a). Therefore, the enhancement of crop yield depends on the introduction of yield-related alien genes to augment the genetic basis. However, the introgression of alien genes is a tedious and time-consuming process (Kumari et al., 2020b, 2020c). Thus, the closest genera of the crop are a more reliable source for gene introduction due to the inhibition of the introduction of alien genes by intergeneric incompatibility, hybrid sterility, and reduced or absent chromosome pairing between alien and crop species and the resulting linkage drag (Bohra et al., 2022). Subsequent generations of hybrids and early backcross progenies exhibited high levels of male and female sterility due to abnormal meiosis (Lelivelt et al., 1993). *In vitro* fusion or protoplast fusion was initially used to introduce genes to overcome the pre- and postfertilization barriers within intergeneric hybridizations that contributed to biotic and abiotic stress tolerance in Indian mustard (Kirti et al., 1992, 1995). Moreover, genetic engineering-induced and spontaneous mutations have also been used to introduce genetic diversity. However, due to the amphidiploid nature of *B. juncea*, the application of reverse genetics to identify mutations that cause quantifiable phenotypic impacts is challenging. Since each phenotype is controlled by at least two mustard homoeologs with presumably redundant activities, combining mutant homoeologs from both subgenomes is necessary to change a monogenic trait (Wells et al., 2014; Emrani et al., 2015). For the trait regulated by an oligogenic or polygenic system in polyploid species, genome editing using CRISPR-Cas9 is not a realistic approach (Endo et al., 2015).

To overcome the consequences of intergeneric hybridization, we used PEG-mediated protoplast fusion to introduce new alleles of *S. alba* into *B. juncea* to avoid potential negative effects such as intergeneric incompatibility and hybrid sterility. Fortunately, we succeeded in achieving the first stable and fertile somatic hybrids of *S. alba* and *B. juncea* with proper meiosis (Kumari et al., 2018; Kumari and Bhat, 2019, 2021). These hybrids were then backcrossed with *B. juncea* as the recurrent parent, resulting in the first backcross progeny with a haploid set of *S. alba* chromosomes with a high degree of gamete viability and subsequent fertility. Among the BC_1_F_2_ generation, we identified a backcross line with the highest number of PBs (26–27) and SB (166) (Kumari et al., 2020b). The shoot branching pattern is controlled at various levels, such as node pattern, meristem determination, and axillary meristem elongation (McSteen and Leyser, 2005). Several genes regulate node patterns in *A. thaliana*, such as *LATERAL SUPPRESSOR* (LAS) (Greb et al., 2003), *SHOOT MERISTEMLESS* (STM) (Long et al., 1996), *REVOLUTA* (REV) (Talbert et al., 1995), and the *REGULATORS OF AXILLARY MERISTEMS* (RAX) genes (Keller et al., 2006; Muller et al., 2006). The inflorescence meristem identity is determined by the floral identity genes *TERMINAL FLOWER1* (TFL1) (Bradley et al., 1997) and *LEAFY* (LFY) (Weigel et al., 1992). Moreover, branch elongation is regulated by several phytohormones, such as auxin, cytokinin, and abscisic acid (Ward and Leyser, 2004). Critical analyses of genes that regulate auxin signaling and transport, such as *AUXIN RESISTANT1* (AXR1) (Lincoln et al., 1990; Leyser et al., 1993; Stirnberg et al., 1999) and *MORE AXILLARY GROWTH* (MAX) (Stirnberg et al., 2002; Sorefan et al., 2003; Booker et al., 2005; Bennett et al., 2006), have shown that these hormones play a central role in branch development.

However, a major challenge was the identification of the genes responsible for the segmental introgressions of *B. juncea* chromosomes, which were introduced in advance BCILs. To address this issue, we developed a set of SSR markers specific to the *S. alba* genome from the draft assembly and used them to genotype core sets of BCILs (Kumari et al., 2020a; Singh et al., 2021b, 2022b). Using a set of 170 monoallelic SSR markers specific to *S. alba*, we identified associated markers with high PB and SB by using genotypic and phenotypic data from two successive years. We utilized association mapping, which is a reliable method for addressing quantitative variations, to better understand introgressed variants (Risch and Merikangas, 1996; Nordborg and Tavaré, 2002; Gupta et al., 2014). The BCILs used in this study have euploid chromosomal counts high fertility, and are stabilized as translocation homozygotes.

Although the size of the introgressed segment likely plays a crucial role in defining BCILs, physical linkage significantly impacts the linkage disequilibrium (LD) between molecular markers (Remington et al., 2001). This provided a genetic basis for the association mapping of genes responsible for a substantial proportion of PB and SB. The extent of LD between linked markers in the complete set of BCILs was notably greater than that between unlinked markers. This study reports the results of association mapping experiments using markers specific to the donor genome and phenotypic data that were subsequently recorded from BC_2_F_6-7_ generations from two successive crop seasons. We identified closely linked markers to pinpoint the genetic regions responsible for PB and SB. The discovery of these markers will facilitate the rapid introduction of the higher branching phenotype into new germplasms, marker-assisted breeding, and selection.

## Materials and methods

### Plant material

We employed a subset of 86 backcross introgression lines (BCILs; BC_2_F_6-7_) of *S. alba*-*B. juncea* somatic hybrids with their parents (*S. alba* and *B. juncea*) for association studies on the number of branches in fully matured plants. These BCILs were derived by backcrossing two stable allohexaploids of *B. juncea* and *S. alba*, namely, H1 and H2, as previously reported by Kumari et al. (2018) and Kumari and Bhat (2019, 2021), with *B. juncea* cv. RLM-198 and NPJ-212 (Kumari et al., 2020b).

### Experimental design and trait measurement

Independent field trials were conducted at the agricultural farm of the ICAR-Directorate of Rapeseed Mustard Research, Bharatpur, India, during the 2021–22 (CS-I) and 2022–23 (CS-II) crop seasons. The experimental field is situated at 77.300°E and 27.150°N. To grow the BCILs, a randomized complete block design was employed with three replications, and standard farming techniques were implemented for mustard without the use of fertilizers or fungicides. The BCILs were cultivated using 45 cm row-to-row and 30 cm plant-to-plant spacing within each row. Five plants at random from each replication of the BCILs were selected to record the phenotypic data, which included the number of PB and SB at the end of flowering.

### Statistical analysis

In this study, we utilized the variability package (https://CRAN.R-project.org/package=variability; Singh and Chaudhary, 1977) in R v4.2.1 to estimate several attributes, including the standard error of the mean (SEm), the critical difference at 5% (CD), broad-sense heritability (H^2^), analysis of variance (ANOVA), environmental variation (EV), genotypic variation (GV), phenotypic variation (PV), coefficient of variation (CV), and frequency distribution among the 86 BCILs.

### Genomic DNA extraction

Genomic DNA was extracted from fresh young leaves of both the parent plants and the backcross progeny (BC_2_F_6_) using a modified CTAB method (Kirti et al., 1995). The extracted DNA was then quantified using a Nanodrop 8000 spectrophotometer (Thermo Fisher Scientific, USA).

### SSR markers

Genotyping was performed using *S. alba* genome-specific SSR markers (Singh et al., 2022b) that were developed using a *de novo* whole-genome assembly (Kumari et al., 2020a). A set of 170 *S. alba*-specific SSR markers randomly chosen from the whole genome was used to genotype 86 BCILs of the BC_2_F_6_ generation, revealing morphological variations in PB and SB.

### PCR and data scoring

The genotyping study used a 10 μl PCR mixture containing 1.6 μl of template DNA (30 ng/μl), 1.1 μl of primer pairs (forward and reverse) (10 mM), 0.30 μl of dNTP mix (10 mM), 1.1 μl of MgCl2 (2.5 mM), 1.1 μl of Taq DNA polymerase buffer (10X), 0.30 μl of Taq DNA polymerase (GCC Biotech, India) (2.5 U), and 4.5 μl of nuclease-free water. The PCR conditions involved initial denaturation for 5 min at 94°C, followed by 40 cycles of denaturation at 94°C/30 s, primer annealing at 57–59°C/40 s, and primer extension at 72°C/45 s, and a final extension at 72°C for 7 min. The PCR products were separated on a 3% agarose gel and visualized using ethidium bromide staining in a gel documentation unit. The amplified bands were scored as present (1) or absent (0) for genotyping analysis (Kumari et al., 2024).

### Population structure and phylogenetic analysis

The population structure of the BCILs was analyzed using the model-based Bayesian clustering method in STRUCTURE v2.3.4. A burn-in period of 10,000 and 100,000 Markov chain Monte Carlo (MCMC) iterations was used, with k ranging from 2 to 10, to investigate the population structure (Pritchard et al., 2000). The ideal number of subpopulations (K) was determined using Evanno’s method through the use of STRUCTURE HARVESTER (Earl and VonHoldt, 2012). In TASSEL v5.0 (http://www.maizegenetics.net/), the genotypic data were utilized to calculate the genetic distance across BCILs, and a phylogenetic tree was constructed using the unweighted pair group method with arithmetic mean (UPGMA) method (Bradbury et al., 2007).

### Kinship coefficient, association mapping, principal component analysis, and linkage disequilibrium

Once the K value was established, the population structure matrices (Q) were identified using STRUCTURE. TASSEL was used to estimate the kinship coefficients (K-matrix) among all genotypes (BCILs) based on similarities in the SSR markers. The association analysis between individual SSR and phenotypic values was conducted at the *p*-value 1e-4 using mixed linear model (MLM) and general linear model (GLM) approaches, combining K and Q matrices (Bradbury et al., 2007). Every marker used for genotyping was employed in the association analysis. A quantile–quantile (QQ) plot was used to compare the relative distributions of the observed and expected -log10(P)-values for each SSR marker–trait association. Principal component analysis (PCA) was used to stratify the population structure. Eigenvalue analysis (Patterson et al., 2006; Price et al., 2006) and a kinship matrix (Malosetti et al., 2007; Pasam et al., 2012) were used to eliminate the effects of structure on the mapping panel and relatedness between the genotypes. To assess linkage disequilibrium, r^2^ was calculated for each allele of the SSR marker using TASSEL software. The calculation was performed using the LD full matrix, with heterozygous calls set to missing. The r^2^ threshold was set at 0.1, so any SSR marker with a r^2^ value below that threshold was considered to have weak LD (Nyine et al., 2019). A graph in the form of an LD heatmap was generated using TASSEL to visualize the distribution pattern of the genome-wide LD decay.

### Candidate gene prediction and annotations

The SSR markers that were found to be associated with branching traits were aligned with the *S. alba* genome. The 40k bp genome sequence located up- and downstream of the SSR was used for complete gene prediction through the AUGUSTUS web server (https://bioinf.uni-greifswald.de/augustus/submission.php), with *A. thaliana* serving as the reference organism. The genes predicted through AUGUSTUS were then annotated using BLASTP with the UniProtKB/Swiss-Prot (Swissprot_v5), nonredundant protein sequence (Nr_v5), and RefSeq protein databases. These predicted genes were also subjected to a local BLASTP for domain searches using CLC Genomics Workbench version 20.0.4 (Qiagen, USA). Furthermore, the PANNZER web server (http://ekhidna2.biocenter.helsinki.fi/sanspanz/) was utilized to predict protein functions (Toronen and Holm, 2022). To further annotate these 47 predicted genes, 1127 branching-related genes in FASTA format were retrieved from the European Nucleotide Archive (ENA). Finally, the predicted genes were subjected to BLASTN with a transcriptome assembly of H1 allohexaploid to confirm their presence in BCILs (Singh et al., 2022a).

## Results

**Figure 1:**
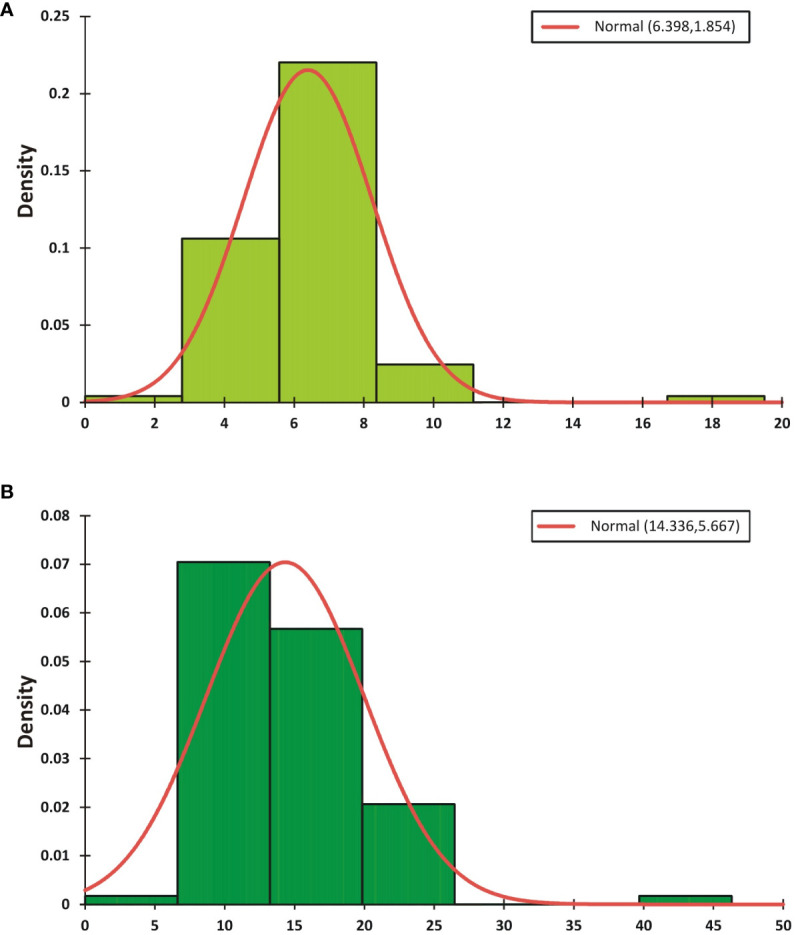
The mean phenotypic distributions of the PB **(A)** and SB **(B)** traits for the 2021-22 and 2022-23 crop seasons. The grand mean value and standard deviation (StDev) are presented in the top right corner of the graph.

**Figure 2:**
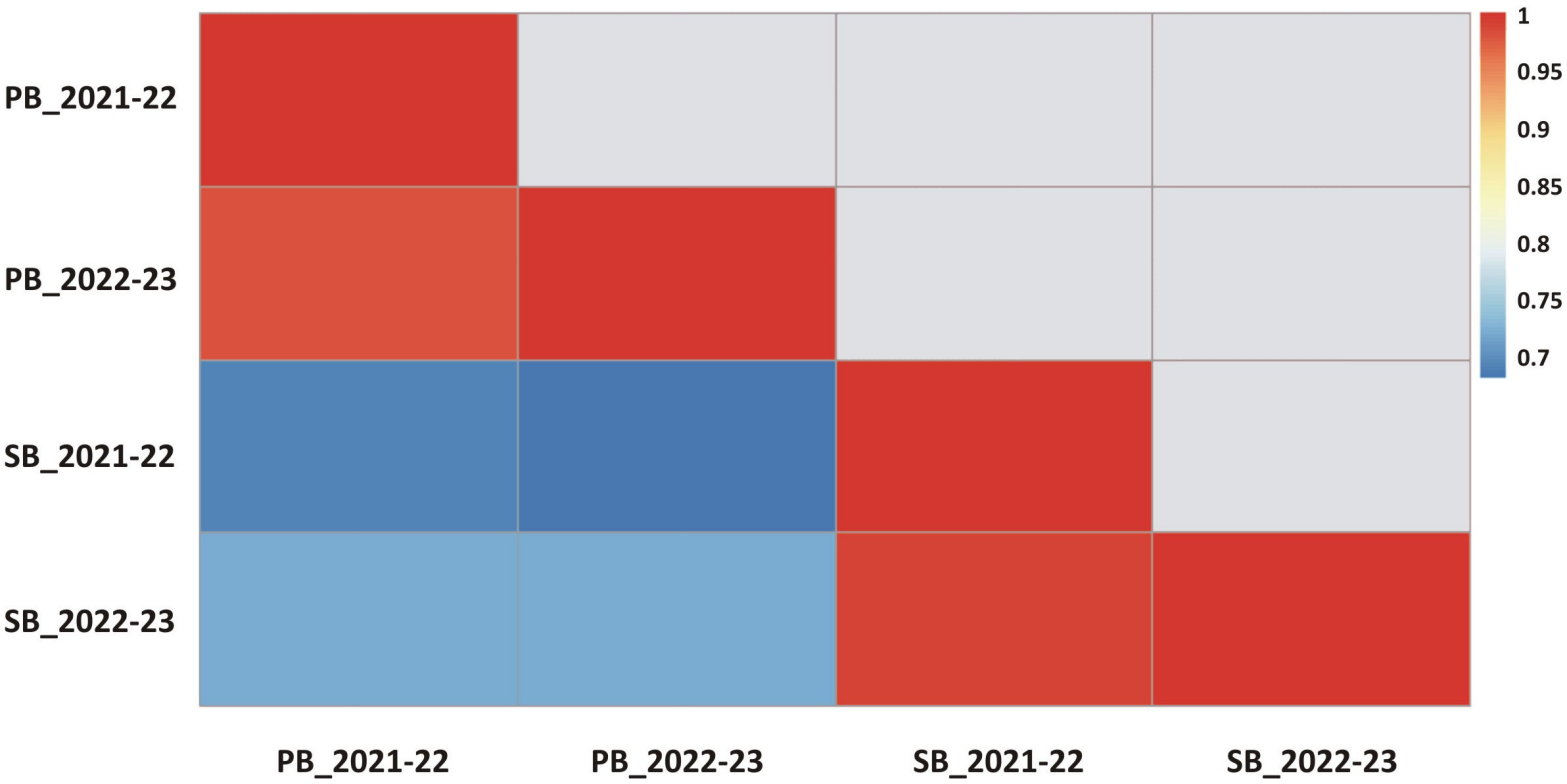
The correlation coefficients between branching traits for different crop seasons are presented (all coefficients were found to be statistically significant at a p value <0.01).

During the CS-I and CS-II crop seasons at the ICAR-Directorate of Rapeseed Mustard Research, phenotypic data were collected from backcross introgression lines (BCILs) of *S. alba* and *B. juncea*. The PB and SB numbers were recorded and showed considerable variability among the BCILs. For example, BCIL 70 had an average minimum of 2.0 PB, while the donor parent *S. alba* in CS-II had an average maximum of 21.0 PB. The grand mean values for the number of PB were 6.32 ± 0.77 and 6.48 ± 0.45 for CS-I and CS-II, respectively. The donor parent *S. alba* had the highest average number of SB at 43.6, followed by BCILs 10 (26.2) and 83 (24), while BCILs 49 (5), 32 (6.6), 28 (7), 66, and 70 (7.2) had the lowest average number of SB. The grand mean values for the number of SB were 14.314 ± 3.06 and 14.36 ± 1.42 for CS-I and CS-II, respectively (**Figures 1A, B**). The cumulative effect of genotypic and environmental variance for both crop seasons resulted in the phenotypic variance of PB and SB, respectively. The phenotypic coefficient of variation (PCV) was greater than the genotypic coefficient of variation (GCV) for both traits, indicating a significant environmental influence. However, both PB and SB exhibited high broad-sense heritability (H^2^) values, suggesting that these traits are not influenced by environmental factors. The genetic variability for these traits was low, with genetic advancement percentages of the mean (GAM) determined to be 36.67 and 51.30 for PB and 36.55 and 69.31 for SB, respectively (**Table 1**). The correlation between PB and SB in both seasons was very high, as shown in the correlation heatmap (**Figure 2**).

**Table 1:** Descriptive statistics of the primary (PB) and secondary branching (SB) traits evaluated in a set of 86 backcross lines (BCILs) of *S. alba* + *B. juncea* allohexaploids.

S. No.	Particulars	PB*		SB*	
		2021–22	2022–23	2021–22	2022–23
1	Maximum	18.0000	21.0000	43.6000	53.0000
2	Minimum	2.6000	2.0000	5.0000	4.0000
3	Grand Mean	6.3221	6.4750	14.3108	14.3614
4	Standard Error of Mean (SEm)	0.7666	0.4454	3.0567	1.4206
5	Critical Difference (CD) 5%	2.1317	1.2389	8.4998	3.9513
6	Critical Difference (CD) 1%	2.8068	1.6315	11.1912	5.2033
7	Environmental Variance (V_e_)	2.9386	0.9919	46.7186	10.0900
8	Genotypic Variance (V_g_)	2.6938	3.3660	20.7626	30.9566
9	Phenotypic Variance (V_p_)	5.6324	4.3579	67.4812	41.0466
10	Environmental Coefficient of Variance (ECV)	26.7372	15.3816	47.9866	22.1182
11	Genotypic Coefficient of Variance (GCV)	25.7372	28.3346	31.9902	38.7419
12	Phenotypic Coefficient of Variance (PCV)	37.2156	32.3403	57.6723	44.6111
13	Heritability (Broad Sense) (H^2^_b_)	0.4783	0.7724	0.3077	0.7542
14	Genetic Advance (GA)	2.3382	3.3216	5.2066	9.9536
15	Genetic Advance as percentage of mean (GAM)	36.6657	51.2988	36.5536	69.3082
16	^$^Kurtosis (Pearson)	15.8060	15.4780	6.8680	6.2890
17	^#^Skewness (Pearson)	2.8770	2.9080	1.7580	1.8440

*Significant at *P* = 0.001; ^$^Kurtosis is the distribution of observed data around the mean; ^#^Skewness is a measure of the asymmetry of the probability distribution of a real-valued random variable about its mean.

### Population structure, linkage disequilibrium, relative kinship, and principal component analysis

**Figure 3:**
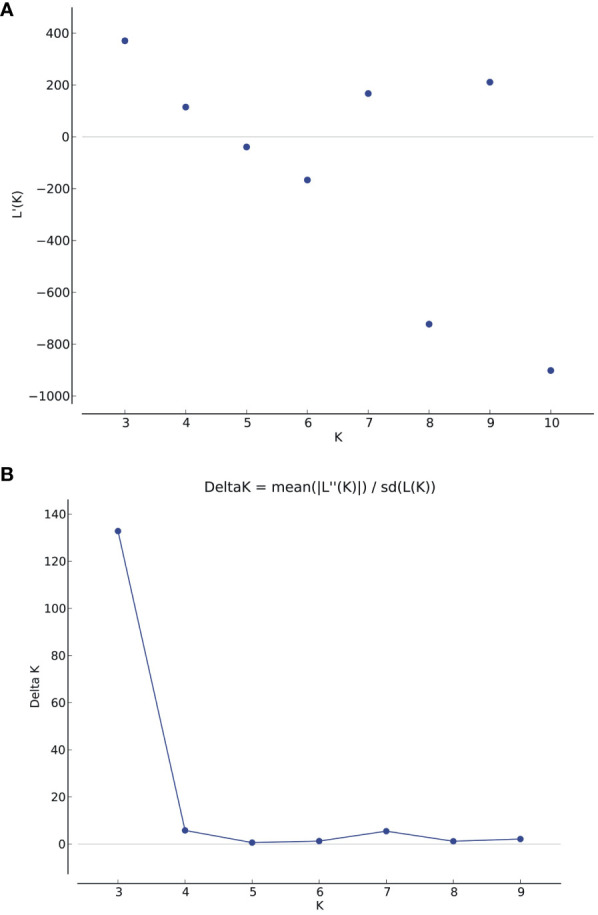
**(A)** The graph displays the estimated logarithm of the probability of data [LnP(D)] for possible clusters (k) ranging from 1 to 10; **(B)** *Delta K* is based on the rate of change in *LnP (K)* between successive *K* values.

**Figure 4:**
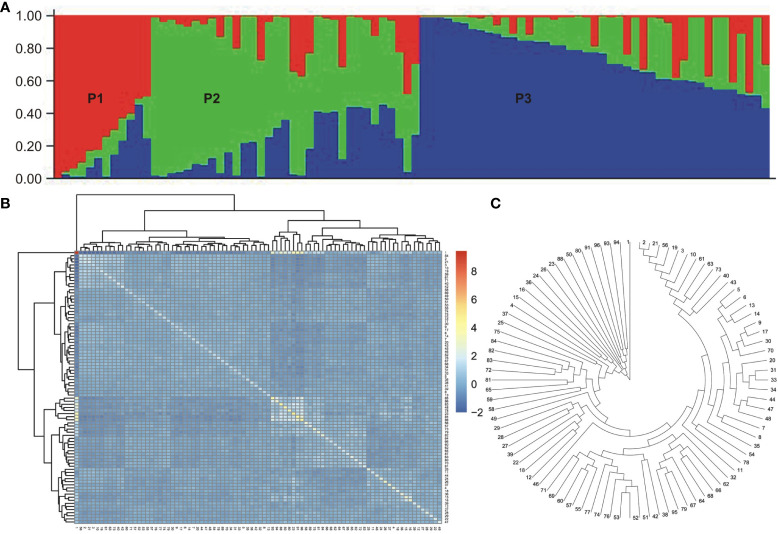
Population structure, Kinship analysis, and UPGMA tree for the mapping panel used in the current study. **(A)** Population structure. Here, different subpopulations (i.e., P1, P2, P3) are indicated using different colors. **(B)** Kinship dendrogram. **(C)** UPGMA tree.

Population structure analysis was conducted on the association panel using 170 donor genome-specific microsatellite markers. Clustering inference was performed with possible clusters (k) ranging from 2 to 10, with five replicates for each k value. The likelihood distribution (LnP k) showed a significant change when k increased from 2 to 3, with the maximum Δk value observed at k=3 (**Figures 3A, B**). The Δk scheme revealed that the 86 BCILs could be grouped into three subpopulations, namely, P1 (donor group; *S. alba*), P2 (mixed population), and P3 (recipient parent group; *B. juncea*). The association panel had 11, 33, and 42 BCILs assigned to the P1, P2, and P3 populations, respectively (**Figure 4A**). These groups were also confirmed through kinship analysis (**Figure 4B**) and UPGMA-based phylogenetic analysis (**Figure 4C**). The phylogenetic tree constructed using the UPGMA method showed three major clades corresponding to the three groups identified by STRUCTURE. BCILs belonging to the mixed population group were distributed between the P1 and P2 subpopulations in the phylogenetic tree. Linkage disequilibrium (LD) analysis and association mapping were conducted using 170 SSR markers in a subset of 86 BCILs. The TASSEL v. 5.0 program was used to calculate LD as the squared allele frequency correlation (r^2^) (Bradbury et al., 2007).

**Figure 5:**
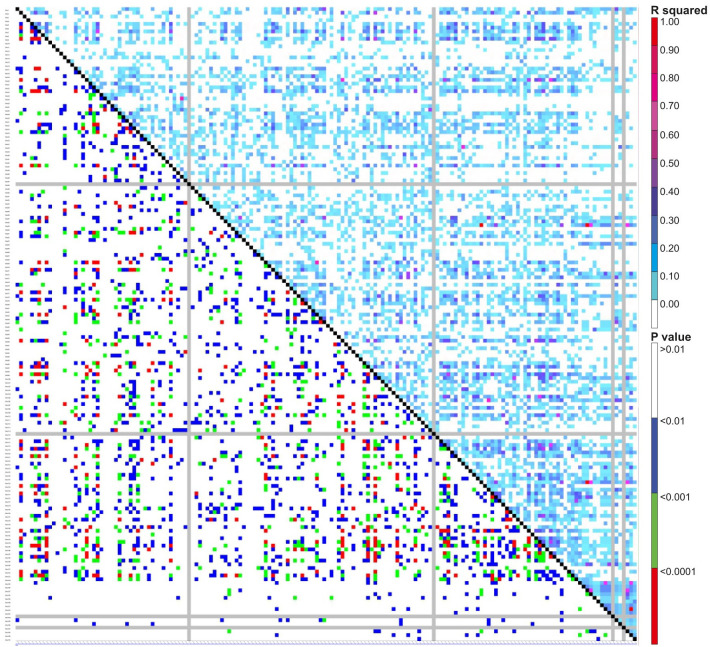
Graphical representation of the LD plot (linkage disequilibrium plot) for the current association mapping panel. Here, the R square and P value are shown as measures of LD (upper and lower diagonal, respectively).

**Figure 6:**
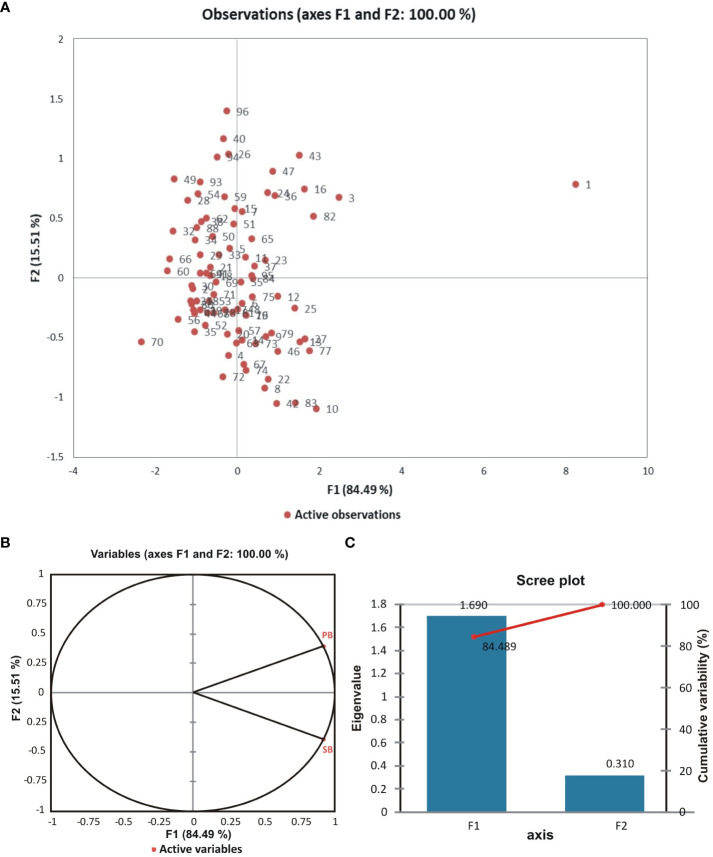
**(A)** Principal component analysis (PCA) between observations (BCILs); **(B)** Circle of correlation between variables (phenotypic traits) after principal component analysis (numbers on the axes represent the correlation coefficient); **(C)** A scree plot showing the eigenvalues of the F1 and F2 factors with their percent cumulative effects on the phenotypes.

Pairwise linkage disequilibrium (LD) was examined in the current genotyping panel using 170 SSR markers to assess the amount of LD at a sliding window size of 50, resulting in the detection of LD in 7225 locus pairs. Among these, 2535 marker pairs (35%) showed substantial LD at the r^2^ threshold of 0.05, while significant LD was observed for 1309 (18%) and 433 (6%) marker pairings at much higher r2 values of 0.1 and 0.2, respectively. The details of both the R^2^ and P values are presented in **Figure 5**. Additionally, principal component analysis (PCA) was conducted to assess the genetic diversity among the association panels (BCILs), with the first two PCs explaining 100% of the genetic variation. The branching trait donor parent was present in the first quadrant, while the recipient plant (*B. juncea*) was present in the fourth quadrant (**Figure 6A**). The correlation circle showed a very strong positive correlation between the PB and SB traits (**Figure 6B**). A total of 87 axes were identified through eigenvalue analysis of the relationships between individual BCILs in the association panel, with the first ten (1–10) principal components accounting for approximately 52% of the variation (**Figure 6C**). The distance matrix was calculated between the BCILs based on their genotyping data, revealing their closeness between 0 and 1, where 0 represented the highest closeness and 1 the greatest distance between BCILs. BCILs 91, 23, 96, and 93 showed distances of 0.435294, 0.458824, 0.464706, and 0.488235, respectively, from the donor parent *S. alba* (Line 01), while BCILs 10 (0.911765), 3 (0.929412), 19 (0.935294), 56 (0.947059), and 21 (0.964706) showed the greatest distances from the donor parent. Additionally, the distance matrix of the recipient parent (*B. juncea*) from the donor parent, *S. alba*, was 1.

### Association mapping for PB and SB

**Figure 7:**
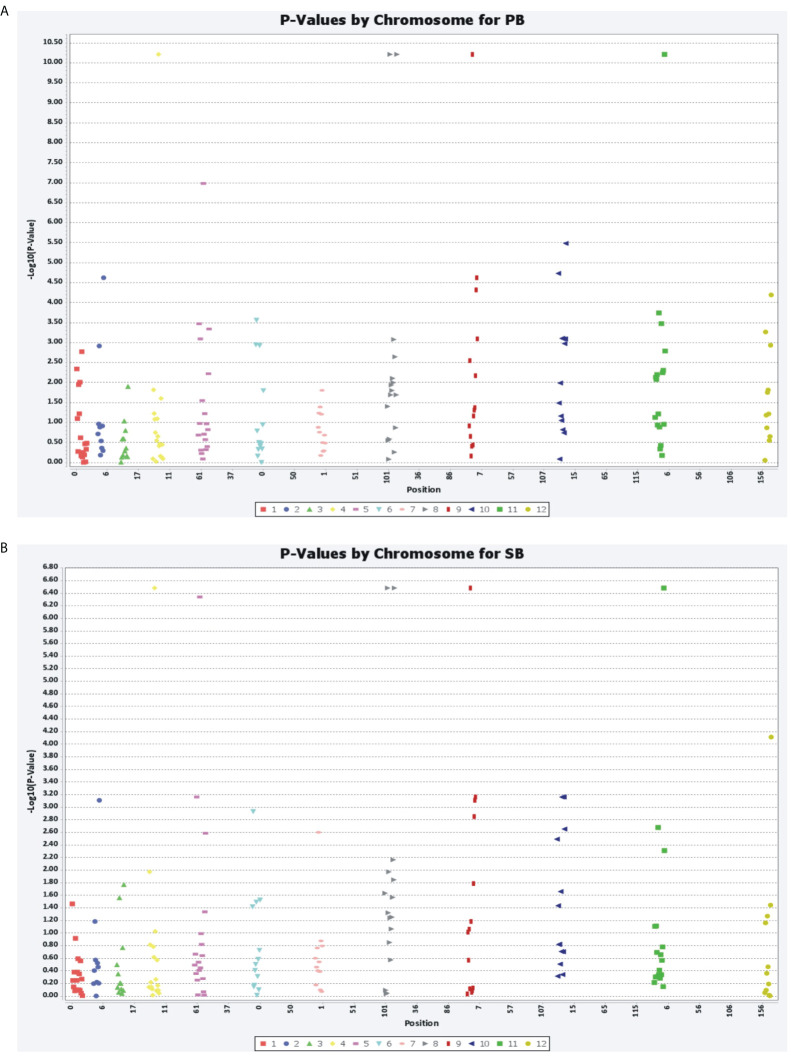
General linear model (GLM) showing the association of SSR markers with PB **(A)** and SB **(B)** traits.

**Figure 8:**
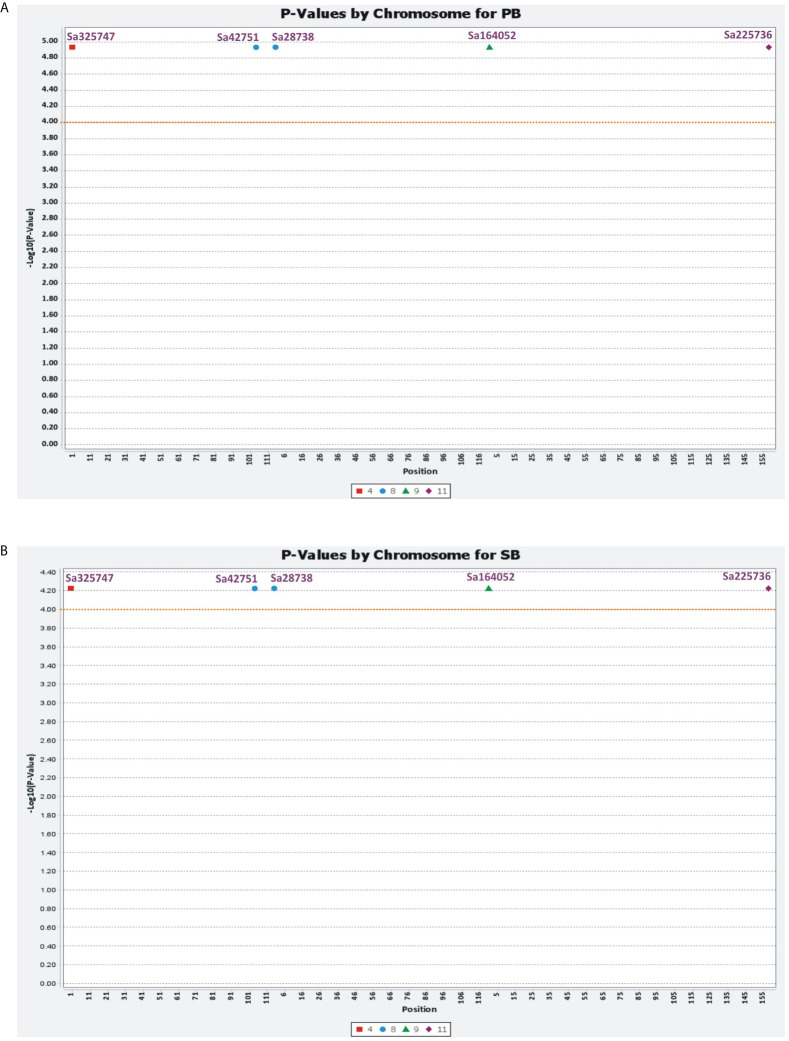
Mixed linear model (MLM) showing the associations of six novel SSR markers with PB **(A)** and SB **(B)** traits.

Marker–trait associations were identified using two different statistical models, MLM and GLM. The MLM used the population structure and kinship matrix (Q + K) model, while the GLM used numerical genotypic and phenotypic data. Five associations (P<0.0001) were identified using MLM for the PB and SB traits, with five common microsatellite markers (Sa28738, Sa42751, Sa164052, Sa225736, and Sa325747) associated with both traits. These markers were distributed randomly in the association panel. These associations were recorded above the threshold value log10(P)= 4.00, with the markers showing an association with the traits at the threshold value 4.934. The total phenotypic variations explained by these markers for PB and SB were 4.55 and 28.95%, respectively. Using GLM, five common microsatellite markers were identified as being associated with the PB and SB traits, which were the same as those identified using MLM. The associations were recorded above the threshold value log10(P)= 6.4, with the markers showing an association with the traits between the threshold values of 6.482 and 10.21. The figures provided more details on the marker–trait associations identified using both models (**Figures 7A, B**, **8A, B**).

### Candidate gene prediction and annotations

To identify candidate genes associated with the branching trait, we utilized the genome sequences of *S. alba* up- and downstream of 20 kb from each microsatellite marker. Our analysis revealed that the 40 kb region surrounding five significant markers contained 47 complete genes. Using the AUGUSTUS web server, we predicted gene sequences for the Sa325747, Sa42751, Sa164052, Sa225736, and Sa28738 markers, resulting in 10, 5, 12, 10, and 10 gene sequences, respectively. We then used BLASTP to identify domains and annotate the putative functions of the predicted genes with protein databases such as the Pfam domain, SwissProt, Nr, and RefSeq (**Supplementary Tables S1**–**S4**, respectively). Among the predicted genes, the majority showed significant similarity to *Arabidopsis thaliana* proteins in all three protein databases, with similarities ranging from 30 to 100%. Interestingly, 35 of the predicted genes showed absolute similarity to branching-related genes (1127 genes) obtained from the ENA database, with 33 genes belonging to *A. thaliana* and 2 genes belonging to *Pisum sativum* (**Supplementary Table S5**).

Our analysis revealed several genes with significant similarity to known regulators of the axillary meristem in *A. thaliana*. For instance, Gene_164052_4 and Gene_225736_1 (At5g23000) were identified as orthologous to *REGULATOR OF AXILLARY MERISTEMS1* (*RAX1*), while Gene_164052_2 (At2g36890) was found to be orthologous to *REGULATOR OF AXILLARY MERISTEMS2* (*RAX2*). Similarly, Gene_28738_4, Gene_164052_6, and Gene_164052_7 (At3G49690) were identified as orthologous to *REGULATOR OF AXILLARY MERISTEMS3* (*RAX3*). We also identified a predicted gene, Gene_42751_2, as orthologous to the *MORE AXILLARY GROWTH4* (*MAX4*) gene (At4g32810), which regulates auxin transport. Other predicted genes, such as Gene_28738_2 and Gene_28738_3 (orthologous to At1G19850), were found to play a role in the establishment of vascular and body patterns during embryonic and postembryonic development. Similarly, Gene_28738_9 and _10 were found to be orthologous to At5g03840, which controls inflorescence meristem identity. Additionally, the predicted genes, such as Gene_28738_5 and _6, Gene_225736_7, Gene_325747_1, and _3, exhibited significant similarity to genes in *A. thaliana* that play an active role in the transcriptional coregulation of *AGAMOUS*. Interestingly, Genes_28738_7 and _8 were found to be orthologous to the *Pisum sativum* gene AAS66906, which regulates shoot branching via physiologically defined mobile signals. Finally, Gene_42751_3 was found to be orthologous to auxin resistance protein 6 (At4g02570), which plays an active role in auxin signaling during embryonic and postembryonic development in Arabidopsis. We also identified Gene_42751_5 as having a role in carbon and nitrogen metabolism and being orthologous to the At1G53310 gene in *A. thaliana*.

This study identified several predicted genes that may be involved in the regulation of plant branching. Gene_164052_12 and Gene_225736_6 were found to be orthologous to *ERECTA1* (*ER1*) (At2g26330), which plays a role in specifying organs that originate from the shoot apical meristem. Gene_164052_8 was predicted to be orthologous to *APETALA1* (*AP1*) (At1g69120), which specifies flower meristem identity and is also required for the normal development of sepals and petals. Gene_164052_9 (At4g08150) was found to be actively expressed in the peripheral and rib zones of the shoot apical meristem but not in the leaf primordia. It is also expressed in the fourth floral whorl, particularly in the cell surrounding the transmitting tissue. Gene_225736_8, orthologous to At3g54720, encodes a glutamate carboxy peptidase and was found to be involved in ethylene-enhanced hypocotyl elongation in light. The alleles of this gene also showed an increased cotyledon number and rate of leaf initiation, along with the transformation of leaves to cotyledons, altered flowering time, and photomorphogenesis.

Gene_325747_5, orthologous to *REVOLUTA* (*REV*) (At5g60690) of *A. thaliana*, has been shown to regulate meristem initiation at lateral positions and is a member of a small homeodomain-leucine zipper family. Gene_325747_6 was orthologous to *AINTEGUMENTA* (ANT) (At4g37750), which is required for the control of cell proliferation and is actively expressed in lateral shoot organ primordia. It also regulates growth and cell numbers during organogenesis and modulates auxin biosynthesis in ovules via the regulation of YUC4. Gene_164052_11 (At1g16410) was found to be orthologous to the *SUPERSHOOT1* (*SPS1*) gene of *A. thaliana* and was significantly associated with branching variation. Gene_SSR325747_2, orthologous to the At3g62980 gene of *A. thaliana*, regulates root and hypocotyl growth, lateral root formation, cell elongation, and gravitropism. Six predicted genes (Gene_225736_3, _4 (At2g45000), _9 (At2g42640), Gene_325747_4 (At2g45010), _7 (At2g42610), and _8 (At2g42650)) were orthologous to *A. thaliana* genes that have putative roles in shoot branching via unknown mechanisms. The remaining 12 genes did not show any similarity with these branching-related genes. Among these 12 genes, Gene_28738_P1 is annotated as an orthologous gene of At5G64730 of *A. thaliana* and is involved in cell wall synthesis, while Gene_42751_P4 (At1G11580) is involved in root growth. Gene_164052_P3 (At5G26742) is involved in chloroplast development, and Gene_164052_P10 (At4G02250) is involved in the regulation of sugar metabolism. These functions were found to be associated with branch initiation and development. Furthermore, all 47 predicted genes were subjected to BLASTN analysis of the coding genes of *A. thaliana*, and the results showed an absolute similarity of 45 predicted genes, while the remaining two genes showed approximately 96% similarity (**Supplementary Table S6**). All the genes predicted in this study were BLASTN with the transcriptome assembly of the H1 allohexaploid, one of the parents of BCILs, and the results showed an absolute similarity of these predicted genes with various coding sequences of the allohexaploid. The successful BLAST results confirmed the presence of introgressed genes controlling the number of PB and SB in the BCILs (**Supplementary Table S7**).

## Discussion

Plant architecture plays a crucial role in determining yield potential, and thus, a positive correlation between high yields and a greater number of PB and SB has been established in Indian mustard (Ehrenreich et al., 2007). Traits that have a large genetic component and a direct correlation with yield, such as the number of branches and pods per plant, number of seeds per pod, seed weight, and flowering time, can be used as suitable selection criteria. Molecular mapping has been used to analyze these morphological characteristics to identify the genes that control them. The yield potential of *B. juncea* has reached a plateau due to changing climatic conditions and genetic uniformity, which are likely to worsen due to various biotic and abiotic stresses, such as insects, diseases, drought, salinity, and high temperature. The expansion of agricultural land is not a feasible solution; hence, increasing crop production through higher yields is essential. The Indian mustard has a narrow genetic base, which makes it difficult to improve its yield potential through interspecific hybridization. Therefore, it is essential to investigate genetic or allelic variability to significantly improve the yield of *B. juncea*. Therefore, alien gene introgression is required to strengthen the genetic base of the crop Brassicas. However, it is challenging to introgress alien genes into crop species due to various barriers, including pre- and postfertilization incompatibility, poor or lack of pairing between chromosomes, hybrid sterility, and consecutive linkage drag (Kirti et al., 1992; Bohra et al., 2022). *S. alba* has been used as a resistance source for biotic and abiotic stresses and seed color for almost four decades (Primard et al., 1988; Hansen and Earle, 1997; Wang et al., 2005; Kumari and Singh, 2019; Kumari et al., 2020c, 2023; Singh et al., 2021a, 2022b). To date, the *S. alba* genome has not been utilized for the introgression of genes related to plant architecture. The identification of alien genes that govern branching phenotypes is also difficult because the introgression of alien genes has still not been reported. However, these genes were identified in the model plant *A. thaliana*, and it has been confirmed that these traits are controlled by multiple genes (Ehrenreich et al., 2007).

Notably, the introgression of alien genes into a crop plant is an intricate and slow process that requires a significant amount of time and effort. To address the issue of poor or lack of pairing between chromosomes of the two species, we used *S. alba* as a donor for genes that govern yield-contributing traits due to its potential for greater branching (Kumari et al., 2011, 2020b). *S. alba* and *B. nigra* (a diploid progenitor of *B. juncea*) share the common ‘Nigra’ lineage of the subtribe Brassicinae (Warwick and Black, 1991; Nelson and Lydiate, 2006), which makes them more suitable for use as protoplast fusion partners of *B. juncea*. In an experiment involving the *S. alba* and *B. juncea* genomes, we recorded approximately 78% genome similarity between the two genera (Singh et al., 2022b). These homologies between the *S. alba* and *B. juncea* genomes might have contributed to the successful production of the first stable and fertile somatic hybrids between *B. juncea* and *S. alba*, with adequate pairing at meiosis, which maintained the fertility of the hybrids and their backcross progenies (Kumari et al., 2018, 2020c; Kumari and Bhat, 2019, 2021). This has also allowed for the inheritance of the bushy plant architecture and higher numbers of PBs and SBs in the backcross generation (Kumari et al., 2018, 2020b; Singh et al., 2023). This, in turn, has contributed to higher yields.

The identification of *S. alba* introgressions in advanced backcross generations has been challenging due to the small size of the chromosomes and the presence of more heterochromatic regions, which is similar to the findings in *Arabidopsis thaliana* (Schweizer et al., 1987). This is because each chromosome in these species has an approximately equal amount of DNA, making it difficult to distinguish segmental introgressions in the recipient genome. Nonetheless, early generations of allohexaploids revealed *S. alba* introgression *via in situ* hybridization, which is a powerful technique for visualizing the localization of DNA sequences on chromosomes (Kumari et al., 2020b, 2020c). With the advanced generation of second backcross progeny of somatic hybrids, these BCILs carry enormous allelic diversity due to the presence of half of the haploid set of the *S. alba* genome, either in the form of segmental or additional chromosomal introgressions. Thus, association analysis by SNP genotyping could not be possible because of sufficient allelic variations within the core set of BCILs. As a consequence, we used a reverse genetics approach in the present study to identify the genes responsible for the greater numbers of PB and SB in a core set of 86 *S. alba*-*B. juncea* BCILs by genotyping with a set of 170 *S. alba* genome-specific SSR markers. However, the chromosome-wide sequence of the *S. alba* genome is not available to date (Kumari et al., 2020a; Singh et al., 2022b). We used these markers as an alternative approach for establishing a marker–trait association to identify the genes that contribute to the greater number of PB and SB in BCILs, which can contribute to greater yields.

The mixed linear model (MLM) and general linear model (GLM) are statistical methods commonly used in association analysis between genetic markers and phenotypic traits. The MLM approach is preferred due to its ability to control population structure and relatedness among individuals, which can lead to false positives in association analysis. The GLM approach, on the other hand, assumes independence among individuals and can produce spurious associations. In this study, both MLM and GLM were employed, and the Q and K matrices obtained from the population structure analysis and kinship analysis, respectively, were incorporated as covariates to account for the confounding effects of population structure and relatedness (Bradbury et al., 2007). It is interesting to note that despite the small size of the association panel, there was wide variation in branch numbers, indicating the presence of natural genetic diversity. The heritability of the trait was also high, suggesting that genetic factors play a significant role in determining the number of PB and SB. The identification of significant marker–trait associations using the MLM (Q + K) model provides further evidence that genetic factors influence the trait. These associations may help in identifying genomic regions or specific genes that are responsible for regulating branch numbers in BCILs.

Using multiple methods to analyze population structure and determine the most appropriate model for association mapping is a common practice in genetic studies. Population structure is responsible for the identification of numerous false-positive QTLs (Zhao et al., 2007). In this study, the Q+K model and the PCA model were considered, but the MLM (Q + K) was found to be the most appropriate for the population of BCILs being analyzed (Yu et al., 2006; Stich and Melchinger, 2009). Additionally, the use of STRUCTURE analysis, a kinship matrix, and a UPGMA tree helped to categorize the BCILs into three populations, which provided valuable information for association mapping analysis. Overall, it is important to carefully consider population structure and appropriate models when conducting association mapping studies to avoid false-positive results and to accurately identify QTLs associated with traits of interest. Indeed, the modification of plant architecture has been a major focus of crop improvement for several years and has contributed significantly to the increase in crop yield during the Green Revolution (Pingali and Raney, 2005).

Based on the MLM (Q + K) model, a total of five significant marker–trait associations were identified for PB and SB in both crop seasons. The genomic regions associated with these markers contained a total of 47 complete genes, 35 of which were identified as candidate genes for regulating the branching trait in BCILs. These genes were identified as orthologous to *AINTEGUMENTA* - ANT (Elliott et al., 1996; Mizukami and Fischer, 2000; Krizek et al., 2020), *APETALA 1* - AP1 (Irish and Sussex, 1990; Byzova et al., 1999; Monniaux et al., 2018), *RAMOSUS*– RMS (Arumingtyas et al., 1992; Grbić and Bleecker, 2000; Foo et al., 2005, 2007), *REGULATOR OF AXILLARY MERISTEMS* - RAX (Keller et al., 2006; Muller et al., 2006), *MORE AXILLARY GROWTH* - MAX (Stirnberg et al., 2002; Sorefan et al., 2003; Booker et al., 2005; Bennett et al., 2006), *MONOPTEROS* - MP (Thomas and Gerd, 1993; Przemeck et al., 1996; Christian and Thomas, 1998), *SEUSS* - SEU (Bao et al., 2010; Gong et al., 2016; Huai et al., 2018), *REVOLUTA* - REV (Talbert et al., 1995), *TERMINAL FLOWER 1* - TFL1 (Bradley et al., 1997), *ALTERED MERISTEM PROGRAM 1 -* AMP1 (López-García et al., 2016, 2020; Yang et al., 2018), *ERECTA* - ER (Godiard et al., 2003; Qu et al., 2017), *SUPERSHOOT1*– SPS1 (Tantikanjana et al., 2001; Ehrenreich et al., 2007), etc. genes that controlling the branching trait. The genes associated with branching traits identified in our study could be utilized in crop improvement programs through targeted genetic modifications, marker-assisted breeding (MAB), and selection (MAS) programs. The introduction of genes responsible for higher branching traits into high-yielding cultivars could lead to increased branching and consequently higher yield potential. This study demonstrated the introgression of 47 genes into *B. juncea* from *S. alba* and identified 35 of these genes as candidates involved in increased PB and SB traits (Ehrenreich et al., 2007). The identified genes were subjected to BLASTN analysis, which matched their sequences with those of the transcriptome assembly of the first stable allohexaploid Brassica (H1) strain developed from RNA-seq at the time of flowering (Singh et al., 2022a). This study highlights the potential for gene introgression to improve crop traits, such as branching, through the transfer of genetic material from related wild species.

## Data availability statement

The original contributions presented in the study are included in the article/**Supplementary Material**. Further inquiries can be directed to the corresponding authors.

## Author contributions

KS: Data curation, Investigation, Methodology, Software, Supervision, Validation, Writing – original draft. PK: Conceptualization, Data curation, Investigation, Methodology, Project administration, Supervision, Writing – original draft, Writing – review & editing. PR: Resources, Writing – review & editing.

## Supplementary material

Supplementary Tables S1–18 BLAST search results for the pfam domain (S1), UniProtKB/Swiss-Prot (S2), Nr (S3), RefSeq (S4), branching genes (BG) downloaded from the European Nucleotide Archive (ENA) (S5), coding gene sequences of *Arabidopsis thaliana* (S6), and coding sequences of the H1 allohexaploid transcriptome (S7) performed to annotate the predicted genes using the CLC Genomics Workbench. Details of SSR markers included in the present study for association mapping (S8).
